# The value of intraoperative dynamic liver function test ICG in predicting postoperative complications in patients undergoing staged hepatectomy: a pilot study

**DOI:** 10.1007/s00423-023-02983-5

**Published:** 2023-07-05

**Authors:** Karoline Horisberger, Fabian Rössler, Christian E. Oberkofler, Dimitri Raptis, Henrik Petrowsky, Pierre-Alain Clavien

**Affiliations:** 1 Swiss HPB Center Zurich, Department of Surgery and Transplantation, University Hospital Zurich, Zurich, Switzerland; 2 Department of General, Visceral and Transplant Surgery, University Medical Center Mainz, Langenbeckstrasse 1, 55131 Mainz, Germany; 3 vivèvis AG – Visceral, Tumor and Robotic Surgery Clinic Hirslanden Zürich, Zurich, Switzerland; 4 Organ Transplant Center of Excellence, King Faisal Specialist Hospital & Research Centre, Riyadh, Saudi Arabia

**Keywords:** Intraoperative ICG, Staged hepatectomy, Post-hepatectomy liver failure, Dynamic liver function test

## Abstract

**Purpose:**

To assess the predictive value of intraoperative indocyanine green (ICG) test in patients undergoing staged hepatectomy.

**Methods:**

We analyzed intraoperative ICG measurements of future liver remnant (FLR), preoperative ICG, volumetry, and hepatobiliary scintigraphy in 15 patients undergoing associated liver partition and portal vein ligation for staged hepatectomy (ALPPS). Main endpoints were the correlation of intraoperative ICG values to postoperative complications (Comprehensive Complication Index (CCI®)) at discharge and 90 days after surgery, and to postoperative liver function.

**Results:**

Median intraoperative R15 (ICG retention rate at 15 min) correlated significantly with CCI® at discharge (*p* = 0.05) and with CCI® at 90 days (*p* = 0.0036). Preoperative ICG, volumetry, and scintigraphy did not correlate to postoperative outcome. ROC curve analysis revealed a cutoff value of 11.4 for the intraoperative R15 to predict major complications (Clavien-Dindo ≥ III) with 100% sensitivity and 63% specificity. No patient with R15 ≤ 11 developed major complications.

**Conclusion:**

This pilot study suggests that intraoperative ICG clearance determines the functional capacity of the future liver remnant more accurately than preoperative tests. This may further reduce the number of postoperative liver failures, even if it means intraoperative abortion of hepatectomy in individual cases.

## Introduction

Post-hepatectomy liver failure (PHLF) is the most serious complication after liver resection and the main cause of death following hepatectomy [1]. Sufficient function of the remnant liver is mandatory for avoiding PHLF. Precise anticipatory assessment of liver function, however, remains a challenge.

The decision as to whether and to what extent hepatic resection can be performed safely is currently usually based on a combination of the preoperative laboratory values, together with volumetric and functional tests. Different diagnostic tools have been developed to assess volume (e.g., calculation of standardized future liver remnant (sFLR)) or function (e.g., technetium-99 m [^99m^Tc] iodide scan, indocyanine green test (ICG)) to predict outcome.

With the careful use of these various tests, the vast majority of patients undergo even extensive liver resections with acceptable morbidity [2, 3]. All tests, however, have limitations, especially in presence of underlying liver injuries, such as those caused by steatosis or preoperative chemotherapy [4]. An intraoperative functional testing would be a logical candidate to identify cases at higher risk of PHLF, irrespective of the fact that their preoperative work-up was unremarkable. Therefore, we postulate that intraoperative ICG clearance could serve as an ultimate reference to prevent PHLF, particularly in complex procedures like the associated liver partition and portal vein ligation (ALPPS), associated with significant morbidity and mortality.

In this pilot study, we performed intraoperative ICG measurements in 15 patients undergoing ALPPS. The main aim was to investigate whether intraoperative measurement of ICG clearance in the future remnant liver could predict postoperative morbidity and liver failure. In a second step, we plan to validate our results in a larger patient cohort.

## Methods

### Study design

From September 1, 2015, to May 30, 2017, we performed intraoperative ICG tests in 15 consecutive patients (aged > 18 years), who underwent ALPPS for secondary liver tumors. The main objective of the study was to analyze a possible correlation between an intraoperative ICG test and postoperative outcome.

Data on demographics; preoperative volumetric and functional tests with magnetic resonance imaging (MRI), computed tomography (CT), and ^99m^Tc iodide scan; preoperative and intraoperative ICG; and postoperative outcome were analyzed. All preoperative tests and analyses refer to the completion operation (step 2 ALPPS). With the exception of one case of extended left hepatectomy, all procedures were extended right hepatectomies. The first step of ALPPS consisted of open portal vein ligation, cleaning of the future liver remnant, and partial (50%) parenchymal transection.

The institutional ethics board of the University Hospital of Zurich reviewed and approved the study protocol (2017–00695). The study was performed according to the Declaration of Helsinki. All patients gave their informed consent to participate in this analysis.

### Indocyanine green test

ICG clearance was measured noninvasively using a LiMON™ device (PULSION Medical Systems SE, Germany). This is a special pulse spectrometer that measures the patient’s blood ICG concentration via finger clip. The patient’s bodyweight ratio (BWR; 0.25 mg/kg) defined the amount to be administered intravenously into a peripheral vein. The results of the ICG test were expressed as the percentage of ICG remaining in the circulation 15 min after injection (R15, %) and the plasma disappearance rate (PDR, %/min). ICG is a water-soluble anionic compound that binds to plasma proteins after intravenous administration. It is selectively taken up by hepatocytes in the first pass and is excreted unchanged in the bile. Thus, ICG clearance measurement reflects the blood flow–dependent clearance, hepatocyte uptake, and biliary excretion [5]. ICG clearance was measured the day before ALPPS step 2 and intraoperatively during ALPPS step 2. During surgery, the ICG measurement was performed immediately after selective arterial inflow clamping of the liver segments to be resected. In all but one case, the portal vein was already ligated in step 1, and the portovenous inflow did not need to be controlled in step 2. In this one case with a one-step procedure, the portovenous inflow was clamped prior to ICG measurement.

### Volumetry

Volumetric data for assessing the sFLR were calculated from the preoperative MRI or CT scans. The FLR volume was expressed as a percentage of the total liver volume. The volumetric cutoff value for safe resection was set at a minimum of 25% for patients with expected healthy liver parenchyma. In patients with known or suspected underlying liver disease, the minimum liver volume was set higher—at least 35%. The required FLR was additionally calculated using the FLR–BWR method, and the minimal FLR volume was required to be at least 0.5% of patient’s weight [6]. To calculate the estimated total liver volume, the sFLR was calculated according to the validated formula by Vauthey et al. [7]. The sFLR, representing the percentage of liver tissue that would remain after resection, was then calculated as the ratio of the FLR to estimated total liver volume [7].

### Hepato-iminodiacetic acid (HIDA) scan

Hepatobiliary scintigraphy with 99mTc-iodide is a quantitative method for evaluating total and regional liver function, including FLR, using radiotracer visualization [8]. The tracer is absorbed by the hepatocytes and subsequently excreted into the bile without any conversion or alteration. Uptake into hepatocytes and intracellular transit are similar to bilirubin, allowing assessment of quantitative liver function [9]. Whether this method is also suitable for assessing liver function in high-risk patients, for example, in patients who require major liver resection, has not yet been conclusively determined. However, de Graaf et al. established a cutoff value for preoperative HIDA examination that was associated with PHLF risk [10].

In our cohort, hepatobiliary scintigraphy was performed in all patients undergoing two-stage hepatectomy. Following de Graaf et al. [10, 11], we used the cutoff value of 2.7%/min/m^2^ to discriminate between normal and decreased FLR uptake rates.

### Postoperative complications

Postoperative complications after ALPPS step 2 were graded according to the validated and severity-oriented Clavien-Dindo complication system [12, 13]. Minor complications were defined as ≤ grade II and major complications as ≥ grade III. Furthermore, we used the Comprehensive Complication Index (CCI®) to assess the cumulative postoperative morbidity [14, 15]. This novel continuous metric model for postoperative complications measures overall morbidity on a scale from 0.0 (uneventful) to 100.0 (death).

PHLF was assessed according to three commonly reported criteria: the International Study Group for Liver Surgery (ISGLS) criteria [16], 50–50 criteria [17], and bilirubin > 7 criterion [18]. The ISGLS criteria are defined as international normalized ratio (INR) and bilirubin above the cutoff value on day 5 after liver resection (we set thresholds of INR ≥ 1.3 and bilirubin ≥ 1.2 mg/dL [≥ 20.4 mmol/L]). Biliary complications were not considered exclusion criteria [16]. The 50–50 criteria are defined as INR ≥ 1.7 (quick 50%) and serum bilirubin ≥ 2.9 mg/dL (50 mmol/L) on postoperative day 5, predicting 50% mortality [17]. The bilirubin > 7 criterion is defined by serum bilirubin levels > 7 mg/dL (119 mmol/L) on postoperative day 5 in non-cirrhotic and non-cholestatic patients and is associated with 90-day mortality [18]. Postoperative laboratory values, including bilirubin, aspartate aminotransferase, alanine aminotransferase, alkaline phosphatase, gamma-glutamyl transferase, creatinine, INR, hematocrit, hemoglobin, and platelet count, were routinely measured from days 1 to 7.

The MELD (Model for End-Stage Liver Disease) score includes bilirubin, INR, serum sodium level, serum creatinine, and the need for dialysis [19, 20]. The MELD score stratifies the severity of end-stage liver disease and is usually used for transplant planning. As it combines liver and kidney function, it was assessed for all patients in our study before and at day 5 after surgery.

### Statistics

We evaluated the baseline characteristics of all patients. The intraoperative ICG test of the FLR was correlated with postoperative blood values, such as bilirubin and INR, and with the CCI®. Intraoperative ICG values in patients with or without major postoperative complications were assessed using comparative analysis. The diagnostic accuracy of intraoperative ICG values for predicting postoperative complications was demonstrated using receiver operating characteristic (ROC) curve analysis. The optimal cutoff point for test positivity was determined with Youden’s index (giving equal weight to sensitivity and specificity), and sensitivity/specificity were calculated. A significance level of 0.05 was used.

No preregistration exists for the here reported study.

## Results

### Characteristics of the study population

Table 1 summarizes the patients’ characteristics. Eleven patients received chemotherapy before surgery. Of these, all were operated on for colorectal liver metastases and underwent a “liver first” approach with ALPPS prior to resection of the primary colorectal cancer. No relevant differences in preoperative liver function, FLR, or hepatobiliary scintigraphy were detected between patients with and without preoperative chemotherapy.

**Table 1:** Patients’ characteristics

	All (*n* = 15)
Age [years]	57 (12) [51–71]
Sex (male/female) [*n*]	10/5 (67%/33%)
ASA II ASA ≥ III	8 (53%) 7 (47%)
Partial ALPPS [*n*]	14 (93.4%)
	• 10 colorectal liver metastases
	• 2 hepatocellular carcinomas
	• 1 intrahepatic cholangiocarcinoma
	• 1 submandibular gland carcinoma
Single-stage hepatectomy [*n*]	1 (6.6%)
	• 1 colorectal liver metastases
BMI [kg/cm^2^]	24 (4)
Time of surgery step 2 [min]	305 (74) [280–378]
Intraoperative transfusions [%]	3 (20%)
Interval between steps 1 and 2 [days]	8 [7–14]
Length of hospitalization [days]	15 (11) [11–25]

Presentation of values as median with standard deviation (round brackets) and interquartile range (square brackets). *ASA*, American Society of Anesthesiology; *ALPPS*, associated liver partition and portal vein ligation; *BMI*, body mass index

Specific postoperative complications are listed in Table 2. Most complications (*n* = 39) were minor. Major complications (*n* = 12) were mostly chest tube placements for pleural effusions and percutaneous drainage of perihepatic fluid collections. Only one patient had ≥ grade IV complications. This patient died 28 days after the second step of ALPPS due to PHLF and septic shock with multi-organ failure. Complications in the interstage period were rare; two patients suffered from minor complications, namely pneumonia and intestinal paralysis. No major morbidity was observed after step 1.

**Table 2:** Incidence of complications by diagnosis and severity

	At discharge	Within 90 days
Any complication in all patients	80%	86.6%
Median CCI [median; IQR]	22.6 (IQR 10.45–41.5)	33.5 (IQR 14.8–56.8)
Minor complications (< III) [*n*]	39 7 ascites – medical therapy 6 antibiotics 6 electrolyte disorders 4 wound infections 4 PONV 3 erythrocyte transfusions 3 gastric tube for paralysis 2 parenteral nutrition 2 delir 1 urinary tract infection 1 acute kidney injury	48 8 ascites – medical therapy 7 antibiotics 7 electrolyte disorders 5 wound infections 4 PONV 3 erythrocyte transfusions 3 gastric tube for paralysis 3 pleural effusion—medical therapies 2 parenteral nutrition 2 delir 2 wound seroma 1 urinary tract infection 1 acute kidney injury
Major complications (≥ III) [*n*]	12 5 chest tubes for pleural effusion 2 drainage abdominal collections 1 PHLF 1 relaparotomy 1 dialysis 1 reanimation 1 reintubation	14 5 chest tubes for pleural effusion 4 drainage abdominal collections 1 PHLF 1 relaparotomy 1 dialysis 1 reanimation 1 reintubation

Each complication was counted, so that multiple complications per patient are possible. *CCI*, Comprehensive Complication Index (measures overall morbidity on a scale from 0.0 (uneventful) to 100.0 (death); *IQR*, interquartile range, listing of minor and major complications in absolute numbers; *PONV*, postoperative nausea and vomiting; *PHLF*, post-hepatectomy liver failure

### Intraoperative ICG measurements and postoperative outcome

**Fig. 1:**
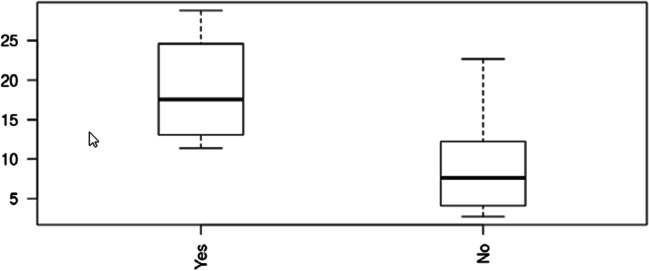
Intraoperative R 15 values of patients with (yes) or without (no) major postoperative complications. Intraoperative R15, median [*r*]: major postoperative complications, 17.5 (IQR 13.9–22.5); minor postoperative /no complications, 7.6 (IQR 4–12.2); *p*-value, 0.049. Values are presented in median with interquartile range

**Fig. 2:**
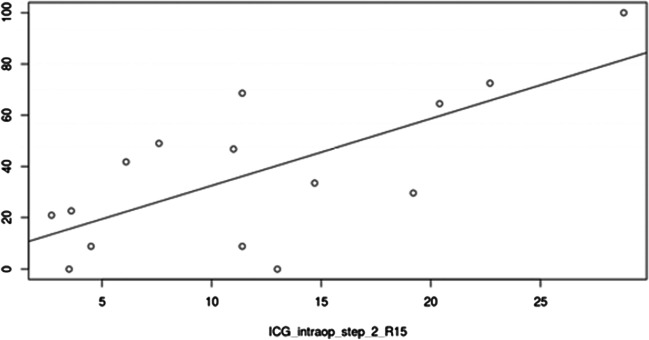
Correlation of intraoperative R15 and CCI 90 days after surgery step 2 (Pearson’s product; *p* = 0.004; estimated correlation: 0.7)

Median intraoperative R15 was 11.4 (IQR 5.3–17) (Table 3). Intraoperative R15 values disclosed a significant correlation with major complications at the end of hospitalization (*p* = 0.049) (Fig. 1), the discharge CCI® (*p* = 0.05), and 90-day CCI® (*p* = 0.0036; Pearson’s product–moment correlation). The estimated correlation between intraoperative R15 and 90-day CCI was 0.7 (Fig. 2).

**Table 3:** Pre- and intraoperative functional measurements

Preoperative R15 [%]	5 [2.2–8.8]
Preoperative PDR [%/min]	20 [16.2–25.4]
^99m^Tc-Iodida [%/min/m^2^]	2.08 [1.8–2.6]
sFLR [%]	43.8 [36.2–54.4]
Intraoperative R15 [%]	11.4 [5.3–17]
Intraoperative PDR [%/min]	14.7 [13.6–20.7]

Preoperative and intraoperative results of liver function tests. Values in median with interquartile range (square brackets). *R15*, retention at 15 min; *PDR*, plasma disappearance rate; *sFLR*, standardized future liver remnant

Median intraoperative R15 was different in patients with and without major complications (18 [IQR 14–22] vs. 8 [IQR 4–12], respectively; *p* = 0.05). Preoperative ICG, volumetry, and ^99m^Tc iodide did not show such correlation to major complications.

Intraoperative R15 correlated significantly with postoperative bilirubin values from days 1 until 7 (all *p* = 0.01 to *p* = 0.03, respectively). Bilirubin showed a correlation to the discharge CCI® at days 4–7 (*p* = 0.005 to *p* = 0.05, respectively) and a significant correlation to 90-day CCI® at days 4, 6, and 7 (*p* = 0.4 to *p* = 0.01, respectively). There was no correlation between intraoperative R15 and postoperative INR.

### Cutoff values

ROC curve analysis revealed that a cutoff value of 11.4 for intraoperative R15 best identifies major complications with 100% sensitivity and 63% specificity. Thus, 11 represents the cutoff point for the intraoperative R15. No patient with R15 ≤ 11 developed major complications during hospitalization, while four out of eight patients with intraoperative R15 > 11 did (*p* = 0.07) (Table 4). Median postoperative bilirubin at day 4 was 9 µmol/L (IQR 8–14) in the R15 ≤ 11 group, and 24 µmol/L (IQR 17–27) in the R15 > 11 group (*p* = 0.072 for non-normal distribution).

**Table 4:** Major and minor complications grouped along the cutoff for intraoperative R15 (*p* = 0.07)

Cutoff intraoperative R15 [%]	Major complications [*n*]	No major complications [*n*]
≤ 11	0	7
> 11	4	4
	4	11

p = 0.07 as outlined in the caption

### Preoperative measurements before ALPPS step 2 and postoperative outcome

Median R15 before ALPPS step 2 or single-step hepatectomy was 5 (IQR 2.2–8.8) and did not correlate with discharge CCI®, 90-day CCI®, postoperative bilirubin (days 1–5), or INR values (days 2–7) (n.s.). Median sFLR was 43.8 (IQR 36.2–54.4); no significant correlation of sFLR was found with R15 (preoperative and intraoperative), ^99m^Tc iodide scan, postoperative laboratory values (bilirubin and INR), or outcome.

Median ^99m^Tc iodide scan was 2.08 (IQR 1.8–2.6) and showed no significant correlation with outcome nor with R15 (preoperative and intraoperative).

### Post-hepatectomy liver failure

Only one patient fulfilled the ISGLS criteria for PHLF [16]. This patient had multiple complications and died due to multi-organ failure and small-for-size syndrome 28 days after hepatectomy. No patient fulfilled the 50–50 criteria [17] or bilirubin > 7 criterion [18]. Median MELD score was 7 (IQR 7–8) and 9 (IQR 8–12) before and at day 5 after surgery, respectively.

## Discussion

The assessment of liver function before major resection is pivotal for the prevention of small-for-size syndrome and postoperative death. In staged hepatectomy, this principle applies to the time before the second (completion) step. Several tools and scores have been established to predict postoperative liver capacity [6, 7, 10, 11, 21], but notably appraisal of FLR function is sometimes inaccurate. While preoperative calculations clearly prevent poor outcomes in most patients, they do not consistently prevent complications in borderline cases [1, 2]. In our study, all patients had excellent functional and volumetric tests prior to embarking in the ALPPS procedure, but several patients subsequently developed major complications after step 2 including one fatality due to PHLF.

There are different reasons for the misjudgment of FLR function. Liver volume does not necessarily correlate directly to function [22–24], and preoperative tests cannot adapt to altered intraoperative circumstances by adjusting for the discrepancy between the planned and actual transection plane [25].

The central finding of our study showed that intraoperative ICG measurement, performed exclusively on the liver portion to be preserved (FLR), precisely correlated with major postoperative complications, even in those patients who showed optimal preoperative test results. For example, intraoperative ICG clearance correlated well with postoperative bilirubin as a surrogate for liver function and clinical outcome. However, neither volumetry nor ^99m^Tc iodide scanning nor preoperative ICG did so.

With complete arterial and venous inflow closure of the part of the liver to be removed (i.e., prior transection), intraoperative ICG measurement selectively informed on FLR, and therefore can estimate its function more accurately than all other approaches of global hepatic measurements. The larger the resection volume (or the smaller the remnant volume), the more vague the preoperative function assessment based on the total volume is. This corresponds to the fact that preoperative ICG test is more exactly suited to minor resections [26]. The 99mTc iodide scan also describes function on a region-by-region basis; however, the 99mTc mebrofenin uptake rate underestimates liver function when serum bilirubin concentration is high (50 μmol/L [3 mg/dL]), as the transport of mebrofenin is dependent from the same transporting polypeptide like bilirubin (organic anion–transporting polypeptide 8; OATP8) and therefore competes with bilirubin [27]. On the other hand, in some patients with rapid hepatic uptake, excretion already starts during dynamic hepatobiliary scintigraphy, hampering the calculation [28].

In terms of major complications, we identified an intraoperative R15 cutoff at 11%. No patient with intraoperative R15 ≤ 11% developed major complications. The cutoff can help in deciding whether to continue the operation, even if the procedure should be stopped for the time being and the interstage period extended. Both the median CCI® at the end of hospitalization and after 90 days were higher in the group of patients with an intraoperative R15 > 11%.

In our cohort, interstage morbidity was exceptionally low with only two minor complications in two patients that did not influence the decision to continue with step 2. This is in contrast to previous findings from Huiskens et al. [29] who showed an overall interstage morbidity of 29%, including 11% complications grade IIIa or higher. The predictive value of interstage morbidity on adverse outcomes after completion hepatectomy had been demonstrated previously [30, 31]. While our study is limited to the ALPPS procedure and not powered for the exact calculation of a cutoff level, the results are in line with the recently reported intraoperative R15 values as an indicator of transient PHLF and other complications after conventional major liver resection [26, 32]. Although not consistent with the common picture of definitive preoperative strategy setting, it is also an opportunity to interrupt vascular dissection at this last possible point to postpone completion of liver resection and thereby prevent complications.

Overall, in terms of comorbidities and indication for surgery, the cohort was homogeneous. Most patients received chemotherapy prior to surgery and underwent the ALPPS procedure for colorectal liver metastases. Since it is known that prolonged preoperative chemotherapy and thus chemotherapy-induced liver injury are significantly associated with PHLF and mortality after liver resection [33], only short chemotherapy with a maximum of eight cycles was administered [34]. Furthermore, we considered an FLR of at least 30% to be appropriate for ALPPS in these patients [35]. However, although 4 out of 5 patients who developed major complications after ALPPS received preoperative chemotherapy, the small sample size of this pilot study does not allow significant correlations to be calculated. In addition, patients with and without preoperative chemotherapy did not differ in terms of preoperative liver function assessments, and no underlying or chemotherapy-induced hepatopathy was documented. It is important to note that the only patient who developed PHLF and died of multi-organ failure after ALPPS had undergone surgery for a large hepatocellular carcinoma without any preoperative systemic therapy. This patient had an intraoperative R15 of 28% during step 2, which was by far the highest value within the cohort. In this case, completion hepatectomy was performed after an unremarkable course over 7 days after the first step. Likewise, the patient did not differ from the rest of the cohort in terms of comorbidities and surgical risk factors. In summary, preoperative ICG before the second step was normal (12.8%), volume gain was adequate (sFLR 17.9 to 36.6%), and the surgery proceeded without complications. Nevertheless, the patient developed PHLF and suffered from numerous complications from which he eventually died 28 days after ALPPS step 2. In sharp contrast to all preoperative measurements, the intraoperative R15 deviated substantially and anticipated the patient’s complication-ridden and ultimately devastating course.

Our study has several limitations. Firstly, only patients with optimal preoperative ICG values underwent completion hepatectomy. Therefore, the analysis focuses exclusively on intraoperative ICG and its correlation with outcome. Thus, a comparison of the predictive value of pre- and intraoperative ICG values was not possible.

Secondly, due to the small number of patients and the one-off occurrence of PHLF, it was not possible to establish a threshold for the prevention of PHLF. The cutoff we have identified does not target the worst possible outcome, but only major complications. Furthermore, the value of intraoperative ICG in patients with cholestasis is unclear. Thirdly, the ALPPS procedures were partial, i.e., only 50% of the parenchyma was transected in step 1. Therefore, residual intraparenchymal porto-portal shunts may have influenced the intraoperative ICG measurements in step 2. However, partial ALPPS has been shown to induce comparable FLR hypertrophy with less morbidity rate than complete ALPPS [36, 37]. The actual effect of such residual porto-portal shunts after partial ALPPS remains unclear. Analysis of possible differences in intraoperative ICG measurements in partial and total ALPPS would be part of a future analysis in a larger patient population.

## Conclusion

Intraoperative ICG cannot be used as the sole decision criterion, but we believe it should be included in the decision-making process. Especially in cases where the preoperative assessment is questionable, patients could be informed that an additional intraoperative ICG measurement is an important complementary tool to clarify the decision for or against completion hepatectomy. In conclusion, the results of this pilot study indicate an advantage of intraoperative ICG measurement over preoperative tests to assess the functional capacity of the future liver remnant. Although these findings are encouraging, the cutoff needs to be validated in larger patient collectives.

## Data Availability

The data that support the findings are not openly available due to reasons of sensitivity but are available from the submitting author upon reasonable request. Data are located in controlled access data storage at USZ.

## Abbreviations

ALPPS: Associated liver partition and portal vein ligation
BWR: Bodyweight ratio
CCI: Comprehensive Complication Index
CT: Computed tomography
FLR: Future liver remnant
ICG: Intraoperative indocyanine green
IQR: Interquartile range
ISGLS: International Study Group for Liver Surgery
MELD: Model for End-Stage Liver Disease
MRI: Magnetic resonance imaging
PDR: Plasma disappearance rate
PHLF: Post-hepatectomy liver failure
ROC: Receiver operating characteristic curve
R15: ICG retention rate at 15 min
sFLR: Standardized future liver remnant
^99m^Tc: Technetium-99 m iodide scan
